# Comparing objective cognitive impairments in patients with peripheral neuropathic pain or fibromyalgia

**DOI:** 10.1038/s41598-020-80740-0

**Published:** 2021-01-12

**Authors:** Henrik Børsting Jacobsen, Tore C. Stiles, Audun Stubhaug, Nils Inge Landrø, Per Hansson

**Affiliations:** 1 The Mind-Body Lab, Department of Psychology, University of Oslo, Gaustadalleen 30, Oslo, Norway; 2 Norwegian National Advisory Unit On Neuropathic Pain, Oslo University Hospital, Oslo, Norway; 3 Department of Pain Management & Research, Oslo University Hospital, Oslo, Norway; 4 Department of Psychology, Norwegian University of Science and Technology, Trondheim, Norway; 5 Institute of Clinical Medicine, Faculty of Medicine, University of Oslo, Oslo, Norway; 6 Clinical Neuroscience Research Group, Department of Psychology, University of Oslo, Oslo, Norway; 7 Department of Molecular Medicine & Surgery, the Karolinska Institute, Stockholm, Sweden; 8 Catosenteret Rehabilitation Center, Son, Norway

**Keywords:** Psychology, Human behaviour, Neurology

## Abstract

Existing studies on cognitive impairments in chronic pain do not investigate peripheral neuropathic pain (PNP) or compare pain conditions in a satisfactory manner. Here we aimed to compare executive dysfunctions in PNP patients with fibromyalgia (FM) and healthy controls (HC). Patients who self-reported cognitive impairments were assessed according to criteria for PNP or FM. Seventy-three patients met criteria and completed testing on executive functioning and IQ measures. We also included twenty matched healthy controls. Regression models controlling for age, sex and IQ, tested associations between group category (PNP, FM or HC) and outcomes. If a substantial association was detected, we followed up with head-to-head comparisons between PNP and FM. Multivariate regression models then tested associations between executive functioning and pain type, controlling for significant confounders. Results from head-to-head comparison between pain conditions showed significant differences on years lived with pain (FM > PNP), the use of anticonvulsants (PNP > FM) and use of analgesics (PNP > FM). When controlled for all significant differences, PNP patients had significantly lower scores on an attention-demanding cued-recall task compared to FM. Poor performance on attention-demanding cued-recall task was associated with PNP, which translate into problems with retaining fast-pace or advanced information.

## Introduction

Executive function is an umbrella term for mental processes enabling us to plan, focus attention, remember, and switch between multiple tasks. Impairments of these functions are prevalent in chronic pain patients^1–4^, and patients rate such impairments as debilitating for their everyday lives^5^.

Current knowledge does not allow for describing differences in executive dysfunction between pain types, as most studies only include heterogeneous, poorly defined pain conditions. A recent review^2^, cited no rigorous comparison of executive functioning between pain types, even though clinical experience argues for substantial contrasts in cognitive difficulties between pain conditions. As an example of this, pain clinicians often claim that fibromyalgia (FM) patients present more problems with executive functioning than those with neuropathic pain (NP)^6^.

Indeed, FM patients do rate cognitive decline and its consequences as one of the most significant limitations to their everyday life^7^, but so do patients with NP^8,9^. However, NP patients reporting cognitive dysfunction receive little attention. As an example, available computerized cognitive training (CCT) programs are based solely on results from patients with musculoskeletal disorders^10,11^, even though potential differences in executive dysfunction between pain conditions is highlighted in these studies.

According to the Neurocognitive Model of Attention to Pain^12^, any executive dysfunction in chronic pain would be the result of the interplay between the attention paid to peripheral input and the goal-directed activity of the brain. NP arises as a direct consequence of a localized lesion or disease affecting the somatosensory system, while FM on the other hand is a centrally driven pain condition^13^. As such, the Neurocognitive Model hypothesizes that there would be differences in executive functioning driven by different inputs to and from the brain^12^, a notion which is yet to be tested in a rigorously classified sample of chronic patients^2^.

Comparing FM and NP patients on executive functioning is also argued from the insight that the former is a pain condition of unknown origin. As such, it lends itself less well to a pathophysiological understanding, potentially driving extended thinking about “why” a FM patient experiences pain. Such extended thinking taxes attentional resources^14^, which over time could drive differences in executive functioning from sustained cognitive activation^12^^.^

Any such difference between pain types would be important as executive functioning is crucial when performing goal-directed behavior and problem-solving^15^, two building blocks of cognitive-behavioral therapy (CBT). Illuminating differences in executive functioning could help evolve a more targeted CBT for chronic pain, potentially improving its therapeutic effects^16,17^. Moreover, as trials of CCT has shown some promise in alleviating impairments^11^, but lack so-called “far transfer” of effects, tailoring condition-specific cognitive training would be a pre-requisite for achieving effect in NP patients^18^.

One meta-analysis has attempted to compare executive functions in pain conditions, indicating significant differences between pain conditions when broadly defining them as FM versus “non-FM” pain^3^. The FM group showed a significant impairment in a composite of executive functioning coined updating, while the heterogeneous non-FM group showed impairments in the executive function composites of response inhibition and cognitive flexibility^3^. While underlining the paucity of studies investigating well-defined pain conditions, these results could be viewed as an indication of differences in executive functioning existing between pain conditions.

Peripheral neuropathic pain (PNP) without concomitant systemic disease would provide a good model when attempting to investigate potential contrasts between etiologically different pain conditions. However, there is a considerable gap in knowledge on how PNP affects cognition. Available studies describe diabetic polyneuropathy^19^, where it is not clear to what extent the dysregulation of insulin and co-morbidities are driving executive dysfunctions^20^. Others mix NP with radicular pain^21^, or report only on a handful of participants with PNP^22–24^.

Only two studies have an adequate sample of PNP patients and these studies use a superficial screening of cognition^25,26^. Moreover, only one study controls for the impact of sleep deficiency^26^, though the impact of poor sleep on cognition is well known^27^. A single comparison of cognitive impairments in neuropathic pain (NP) and generalized pain (including FM) exits, using neuropsychological tests of verbal memory and inhibition^28^. However, this study mixed central and peripheral NP, had a small sample size (n = 14), and no healthy control group. Nevertheless, a higher percentage of FM patients than NP patients performed below cut-off for normal performance on the interference and switching conditions of the Stroop test. Conversely, more NP patients compared to FM patients performed below cut-off on the California Verbal Learning Test^28^. This result aligns with a review claiming that NP could specifically impact the most attention-demanding cognitive processes, such as holding information in working memory for long-term encoding and retrieval^2^.

As both a review, clinical experience, and a single experimental study, indicates differences in executive functioning between pain conditions, the aim of the current investigation was to provide a detailed description of executive functioning in PNP and compare this to FM. As a vantage point for our comparison we chose the Unity and Diversity Model (UDM) of executive functioning, which indicates three core components of executive function that are functionally separable^29^.

These components are inhibition (inhibitory control and interference control), updating or working memory (the ability to maintain accurate representations of information which changes over time) and cognitive flexibility (switching attention from one source to another and monitoring current internal and external states)^30,31^. Results from twin studies show that the correlations among the three components are substantial, but far from perfect (~ 0.5)^32^. Since both a previous study^28^ and a recent review^2^ suggested that patients with NP could have a specific impairment of executive functioning when processing more attention-demanding recall tasks, an appropriate test should be added when testing these chronic pain patients.

Moreover, in studies of heterogeneous chronic pain conditions, insomnia worsens working memory performance^33^, and both patients with FM^34^ and NP^35^ report higher levels of insomnia severity when compared to other pain conditions. In addition, both depression and pain medication can influence executive dysfunction and need to be controlled for^3^.

We here aimed to evaluate whether there were differences between FM, PNP or healthy controls (HC) on four tests of executive functioning. Three of the tests reflect the core components in the UDM of executive functioning^29^, and the fourth tested attention-demanding cued recall. Our specific hypotheses were that (1) FM patients would demonstrate executive dysfunctions beyond HC and patients with PNP on the three executive functions of inhibition, updating and flexibility; (2) that PNP patients would perform significantly worse on an attention-demanding cued-recall task compared to FM patients and HC.

## Methods and materials

### Setting

From July 2016 until March 2018, we recruited potential candidates, prospectively and retrospectively, from the patient population at the Department of Pain Management and Research, Oslo University Hospital. This is a tertiary multidisciplinary pain clinic and all pain conditions would have lasted more than 3 months when referred to the clinic.

In addition, we circulated information leaflets to all relevant pain clinics and patient organizations asking general practitioners to refer patients to the study. Potential patients had to indicate that they experienced problems with memory and/or ability to concentrate. Finally, twenty HC were recruited through a hospital network of potential volunteers after the inclusion of patients was completed, in order to match HC with patients on age and sex.

**Figure 1:**
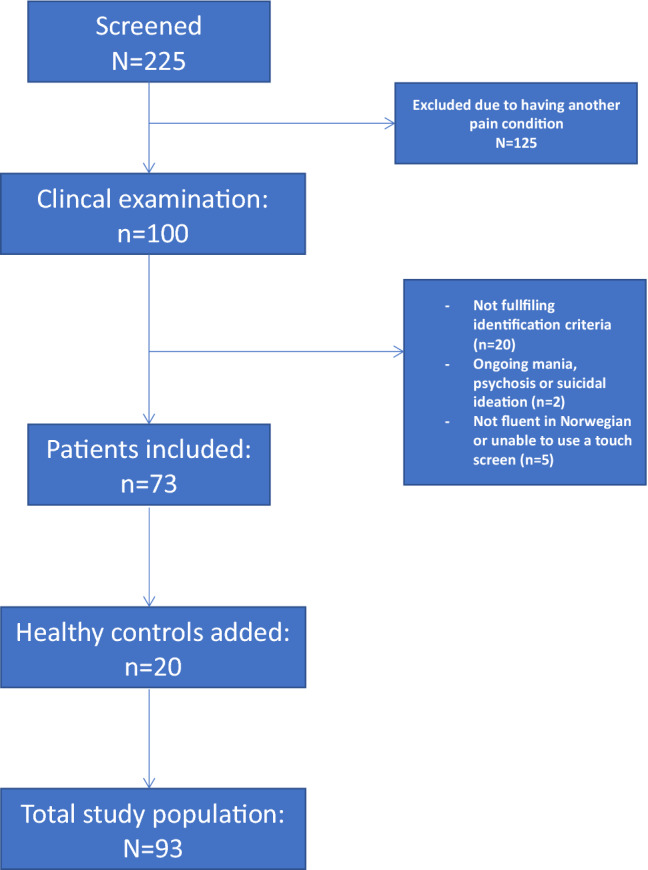
Flow of participants throughout the study from screening to inclusion.

We examined 225 patients, and 79 patients met the criteria for inclusion in the study. Of these patients, 73 initiated and completed neuropsychological testing and were available for the current analyses (Fig. 1). In addition, twenty healthy controls were added to complete the data collection. We then collected data from self-report forms as well as performed neuropsychological testing at a clinical visit. HC participated in identical IQ and neuropsychological testing, but did not complete any questionnaire.

Patients completed several questionnaires upon arrival at the clinic using a tablet connected to an online registry system (OPR)^36^. We then performed IQ testing and neuropsychological testing within 30 min of finishing the online survey. The complete testing regime lasted a total of 2.5 h per patient.

We alternated the sequence of neuropsychological tests several times during the data collection period to ensure that no sequential effects were confounding the results. Before the initiation of testing, participants filled out visual analogue scales (VAS) indicating level of anxiety and if they had a desire to leave the situation. The VAS was presented as a straight horizontal line of fixed length (100 mm) across a continuum from none to an extreme amount of anxiety or avoidance orientated from the left (none) to the right (extreme). Sleep parameters were recorded immediately and for seven consecutive days at home after collecting baseline data.

### Ethics

The Regional Committee for Medical Health and Research Ethics in South-Eastern Norway approved this study (approval number 2016/595) and the protocol was published on www.dam.no (grant number: 2016/FO78689). In the protocol and in ClinicalTrials.gov it was registered as study 1 in a larger randomized controlled trial (ClinicalTrials.gov Identifier: NCT02824588, registered 06/07/2016). We here confirm that the study complied with good clinical practice, including the most recent version of the declaration of Helsinki, as well as all relevant rules and regulations in Norway. Participation was based upon signed informed consent, and thorough information was provided orally as well as in writing.

#### Inclusion and exclusion of patients

The patients’ pain conditions should have lasted longer than 3 months, i.e., regarded as chronic, when referred to the pain clinic. At the clinic, the patients filled out a standardized questionnaire before a multidisciplinary pain team (physician, psychologist and occasionally a physiotherapist) evaluated them.

In cases of suspected PNP or FM, the patients were referred within the clinic to a specialist, either a neurologist (PNP, author PH) or one specific specialist in physical medicine and rehabilitation (FM), who then performed a structured assessment of the disorder. The following two independent levels of criteria were needed to arrive diagnostically at definite *peripheral neuropathic pain*: First, the participant had to (1) present pain with a distinct, neuroanatomically plausible distribution, and (2) a history suggestive of a relevant lesion or disease affecting the peripheral somatosensory nervous system. Then, during examination the participant had to (3) demonstrate a distinct neuroanatomically plausible distribution of somatosensory disturbances and (4) harbor a relevant lesion or disease by at least one confirmatory test (e.g., electrophysiological abnormalities, surgically induced injuries)^37^.

The following criteria needed to be met to arrive at a diagnosis of *fibromyalgia*: The participants had to present: (1) pain in the left side of the body, pain in the right side of the body, pain above the waist, and pain below the waist. In addition, axial skeletal pain (cervical spine or anterior chest or thoracic spine or low back) had to be present. In this definition, shoulder and buttock pain is considered as pain for each involved side. (2) Participants’ had to describe pain in 11 of 18 tender point sites on palpation during a clinical examination (reumatology.org;^38^). A confirmed condition of either at least probable peripheral neuropathic pain^37^ or fibromyalgia was necessary in order to be included in the study.

Exclusion criteria were reviewed after the aforementioned examinations had been completed. A diagnosis or suspicion of ongoing mania, psychosis or suicidal ideation with previous suicidal attempts was considered a cause for exclusion. Participants were also excluded if suicide attempts or plans of suicide were reported during the project period.

It was also an exclusion criterion if participants could not speak fluent Norwegian, were pregnant or unable to use the touch screen used for neuropsychological testing.

Finally, participants were excluded if they had a combination of PNP and FM, or if they had any disorder or diagnosis that could otherwise explain any potential cognitive impairment, such as an unrelated stroke or diabetes. Chronic pain participants often use medications, so we allowed for stable analgesic medication throughout the test period.

### Executive functioning

All participants completed four tests from Cambridge Automated Neuropsychological Test Battery (CANTAB). CANTAB is based on self-administrated neuropsychological tests, and is a widely used cognitive assessment tool (for an overview see e.g.,^39^). The tests were presented on touchscreen Windows 7 tablet PC running CANTAB-eclipse software. The order of the tests was alternated three times during the study period.

The tests and outcomes are described in detail below and relate to the three functional areas described in the non-unitary model of executive functions^31^, as well as an outcome relating to attention-demanding cued recall. A detailed description of each outcome is listed in the appendix.

*The executive component of inhibitory control* was measured with the Stop Signal Task (SST). The SST is a response inhibition test that measures a subject’s ability to inhibit a pre-potent response. The outcome variable used in the study was an estimate of the stop signal reaction time (SSRT) in milliseconds (lower results indicate higher performance). This outcome provides a measure of the speed of the inhibitory process^40^.

*The executive component of updating* was measured through the Spatial Working Memory (SWM) task. This task assesses spatial working memory by measuring a subject’s ability to retain spatial information and to manipulate remembered items in working memory. The pre-selected outcome was a component score reflecting the strategy participants´ used to avoid unnecessary errors.

*The executive component of flexibility* was measured using the Intra-Extra Dimensional Shift (IED) task. The IED is a computerized analog of the widely used Wisconsin Card Sorting Test and is a test of cognitive flexibility. The pre-selected outcome was IED total errors, a composite of the number of completed stages and the number of errors made.

*Attention-demanding cued recall* was measured using the Paired Associates Learning (PAL) task. The PAL task is a cued recall test that assesses memory and new learning. The pre-selected outcome was performance on the hardest stage, total errors 8 shapes adjusted, which report the numbers of errors made on the last, most difficult stage of the PAL test.

#### Medication

Participants reported daily medication usage upon inclusion and this was controlled with the list of medications provided by the general practitioner and any information given during the interview by the pain specialist.

#### Sleep deficiency

Each participant wore the Philips Respironics Actiwatch Spectrum or Spectrum Pro on the wrist for 7.5 days following the visit to the department. In addition, patients filled out a sleep diary for the same time-period yielding a comparable sleep entry should the actigraph data for some reason not reflect a valid sleep pattern. The Actiwatch and sleep diary were used in combination to create the variables *sleep efficiency* and *average total sleep time*.

#### Intelligence testing

The Wechsler Adult Intelligence Scale (WAIS) IV^41^ measures intelligence in adults. It consists of four index scores attempting to measure four major components of intelligence. As two of the components are strongly correlated with CANTAB tests, namely working memory and processing speed, we chose to use the two components that would add the most to the examination of intelligence in addition to our tests of executive functioning. These were the indexes of verbal comprehension and perceptual reasoning. In the current study, we chose to use the subtests of similarities and matrix reasoning to measure the corresponding indexes^41^.

#### Depression

Patients admitted in the study had been diagnosed by their general practitioner with a musculoskeletal (L), mental (P), or general/unspecific diagnosis, e.g., fatigue, burnout (A), (the International Classification of Primary Care (ICPC) (2nd edition). To determine the presence or absence of *depression* we used the GP referral where a depression was indicated by an ICPC-2 diagnosis as well as a self-report measure of mental distress included in the OPR.

### Patient reported variables

A complete list of the measures in this study has been published previously through a detailed description of the online registry (OPR)^36^. Here we give a brief account of selected variables included in the analyses for this study: *Usual pain intensity* (0–10); *Pain bothersomeness* (0–10; *Chalder Fatigue Scale*^42^; *Work status;* A modified *Oswestry Disability Index* (ODI);^36^; *Insomnia Severity Index* (ISI).

### Statistical analyses

Demographics and patient characteristics were analyzed as either number and percent, or mean and standard deviation (SD). We performed bivariate correlations to check for substantial correlations between the chosen executive functioning measures and potential covariates before moving on to regression models.

The regression analyses in the current study were performed in two stages. In the first stage, we wanted to detect significant differences in the four executive function outcomes between categories FM, PNP and healthy controls, while controlling for age, sex and IQ.

We therefore performed four hierarchical linear regression models, where the variables were entered in steps, using the four selected outcomes as dependent variables in the four models. In all regression models, we entered age and sex in the first step to control for any inherit differences as both age and sex could affect executive functioning (1), in the second step (2) we entered verbal and performance IQ to control for any differences in intellectual ability. Then in the third and final step (3), we entered the categorical group variable using controls as the reference value (FM, PNP and healthy controls).

If the subsequent regression output showed a significant difference between categories (FM, PNP and healthy controls) controlled for age, sex and IQ in this initial stage, we aimed to move on to the second stage of regressions.

Through hierarchical linear regression, we wanted to evaluate and test for substantial differences between the two categories of pain, now excluding healthy controls. Because we aimed to evaluate differences between pain categories, this next stage of regressions was to be performed while controlling for all other significant differences between the two pain types, while keeping age, sex and IQ in the model. Given the interval or categorical nature of the variables investigated, independent *t* tests or chi square statistics were used to test for group differences between FM and PNP on self-reported registry variables, sleep deficiency and medication. Significance level to detect substantial differences was set at *p* < 0.05.

Again, we used a hierarchical linear regression where we entered age and sex in the first step (1) and in the second step (2) we entered verbal and performance IQ. In the third step (3) we entered years lived with pain. In the fourth step (4), we entered the categorical use of analgesics yes (reference category) or no. In the fifth step (5) we entered use of anticonvulsants yes (reference category) or no, before the sixth (6) and final step where pain condition (FM vs PNP) was entered using FM as the reference category. This stepwise approach allowed for investigating changes in explained variance when introducing the diagnostic category, while controlling for other significant differences.

All statistical analyses were performed using SPSS version 25.

## Results

The participating pain patients in this study were predominantly females (73%), married (44%), with high school education (49%), who were currently out of work (59%). The healthy controls were matched on age and sex. None of the participants reported anxiety for the test being administrated, or a wish to avoid the neuropsychological testing as indicated on VAS before administering the test.

Table 1 shows the absolute difference in performance between the three groups on the chosen CANTAB outcomes. The FM group had on average a longer response time on the inhibition task than PNP patients and healthy controls, indicating a reduced inhibitory control. On the cognitive flexibility task, there were only small differences between groups.

**Table 1:** Four theoretically selected outcomes from the CANTAB test battery were used as executive functioning tests in patients with confirmed fibromyalgia (FM) or peripheral neuropathic pain (PNP) or healthy volunteers (HC). The test results are presented as mean and standard deviation (SD).

	PAL total errors 8 shapes	SST reaction time last half	IED Total errors	SWM Strategy
FM(N = 44)	12.30 (SD = 9.15)	220.63 (SD = 52.24)	30.34 (SD = 34.19)	34.07 (SD = 6.17)
PNP (N = 29)	16.38 (SD = 11.22)	200.76 (SD = 49.21)	35.00 (SD = 49.18)	31.03 (SD = 6.83)
HC (N = 20)	12.20 (SD = 10.93)	189.60 (SD = 39.31)	32.50 (SD = 34.80)	31.80 (SD = 5.34)

On the working memory task, the FM group showed a higher score on the use of working memory strategy than both the PNP group and healthy controls. The PNP group on the other hand had more errors on the cued recall task when this task reached the hardest level. The results show that the participants with PNP made more mistakes when asked to remember shapes hidden in eight different boxes (see Table 1 for details).

When testing for any substantial contribution of pain category on the selected outcomes, we investigated the difference between categories (FM, PNP and healthy controls) on performance on neuropsychological tests while controlling for age, sex and IQ. Only the outcome from the PAL test showed a significant difference between categories (PAL 8 shapes, *p* = 0.04), when controlled for these covariates (see Table 2 for details). As none of the other outcomes had significant associations with pain category, the data from the other three regression models are only included in the appendix (Table A2, A3 and A4).

**Table 2:** The executive functioning outcome PAL analyzed using a multivariate linear regression model. The category and its association with this outcome were controlled for age, sex IQ and group categories (FM, PNP and HC). These variables were then added in steps to investigate r^2^ change per step.

PAL 8 shapes total errors					
Steps		Exp B (95% CI)	*p* value	T value	R^2^ change
1	Age	0.08 (− 0.19,0.34)	0.56	0.59	0.06
	Sex	0.45 (− 6.10,6.99)	0.89	0.14	
2	Age	0.07 (− 0.20,0.34)	0.63	0.49	0.16
	Sex	0.64 (− 5.53,6.81)	0.84	0.21	
	Verbal IQ	− 0.73 (− 1.32, − 0.15)	0.02	− 2.51	
	Performance IQ	− 0.32 (− 0.99,0.36)	0.35	− 0.93	
3	Age	0.09 (− 0.17,0.36)	0.49	0.69	0.06
	Sex	− 1.01 (− 7.22,5.20)	0.75	− 0.33	
	Verbal IQ	− 0.75 (− 1.32, − 0.18)	0.01	− 2.62	
	Performance IQ	− 0.30 (− 0.96,0.36)	0.37	− 0.90	
	Group (HC is ref; FM:2 PNP:3)	5.38 (0.18,10.39)	0.04	2.07

**Table 3:** Demographics, patient reported outcomes and medication usage^a^ are presented in 73 patients with either Fibromyalgia or Peripheral neuropathic pain (included from 2016–2018). Variables are categorical, cross tabulated and tested with a Chi Square significance test between the two different pain conditions.

Categorical variables	Fibromyalgia	Peripheral neuropathic pain	Chi sq
	N (%)	N (%)	
**Sex**			*p* = 0.01
Men	5 (11)	11 (39)	
Females	40 (89)	17 (61)	
**Civil status**			ns
Single	7 (18)	5 (22)	
Married/co-inhabitant	23 (61)	17 (74)	
Divorced/widowed	8 (21)	1 (4)	
**Education**			ns
Primary/secondary school	2 (5)	2 (8)	
High school diploma	20 (51)	11 (46)	
College/university less than 4 years	14 (36)	11 (46)	
College/university 4 years or more	3 (8)	0 (0)	
**Work status**			ns
Not working	23 (59)	11 (46)	
Working	16 (41)	13 (54)	
**Comorbid diagnoses**			ns
Depression	5 (11)	6 (21)	
**Medication usage**			
Opioids	9 (21)	10 (35)	ns
Anticonvulsants	9 (21)	14 (48)	*p* = 0.02
No medication	23 (54)	7 (24)	*p* = 0.05

^a^N does not equal PNP = 28 or FM = 43 on all variables due to missing data, or participants responding with “not applicable”.

**Table 4:** Demographics and patient reported outcomes are presented in 73 patients with either Fibromyalgia or Peripheral neuropathic pain (included from 2016–2018). Variables are interval and tested with a *t* test to determine significant differences between the two pain conditions.

Interval	Fibromyalgia	Peripheral neuropathic pain	*t* test
	Mean (SD)	Mean (SD)	
Age	48.5 (10.9)	45.6 (12.3)	ns
Pain intensity^a^	6.8 (1.6)	6.5 (1.9)	ns
Pain bothersomeness^a^	7.0 (1.7)	7.2 (2.1)	ns
Years lived with pain	17.0 (11.6)	5.5 (7.1)	*p* < 0.01
Mental distress (0–4)^a^	2.1 (0.4)	2.2 (0.5)	ns
Insomnia severity (0–28)^a^	13.7 (6.1)	16.0 (7.2)	ns
Oswestry Disability index (0–50)^a^	34.78 (11.02)	34.2 (11.0)	ns
Fatigue (0–11)^a^	8.06 (3.0)	7.2 (3.4)	ns
Verbal IQ	22.8 (5.1)	22.7 (4.5)	ns
Performance IQ	17.6 (4.8)	17.5 (4.9)	ns
Sleep efficiency %	80.6 (8.93)	79.1 (8.20)	ns
Averaged sleep (hours)	6.9 (1.48)	7.3 (1.27)	ns

^a^Higher is worse.

We then investigated any significant differences of the selected covariates in order to include potential confounders in our regression model. Categorical demographic variables are presented in Table 3 by condition entity (FM or PNP). Interval demographic variables for FM and PNP are presented in Table 4.

To evaluate substantial differences between the two pain categories on PAL 8 shapes, we then excluded healthy controls from the analysis and looked for significant differences between pain types. When tested with a chi-square statistic for categorical variables, the two groups had significant differences in sex, medication usage and use of anticonvulsants (*p* < 0.05). None of the other categorical variables showed substantial differences between the two groups.

*T* tests of statistical difference showed that the FM group on average had lived with pain significantly longer than the PNP group (*p* < 0.05). Otherwise, the *T* tests showed no significant differences between the FM and PNP groups on pain intensity or bothersomeness, IQ measures, mental distress, quality of life, fatigue or sleep parameters.

To test the association of pain category we then added the significant differences between pain types as covariates in the final model. A hierarchical linear regression models showed that when controlling for age, sex, IQ, years lived with pain, taking any analgesic, and taking anticonvulsants, having peripheral neuropathic pain remained significantly associated with the number of errors made on the hardest stage of the PAL test (PAL 8 shapes, *p* = 0.02). Adding pain category in the final step resulted in an r^2^ change, which explained 9% of the variance on the dependent variable.

Details of the final step (step 6) of the hierarchical regression analysis is depicted in Table 5, while the other steps are included in the appendix.

**Table 5:** The executive functioning outcome PAL analyzed using a multivariate linear regression model. The condition category and its association with this outcome were controlled for age, sex, IQ and all other variables showing significant differences between categories. These variables were then added in steps to investigate r^2^ change per step. The final step, step 6, shows all variables in the model and how they affect the chosen outcome, as well as r^2^ from adding condition category.

PAL 8 shapes total errors				
	Exp B (95% CI)	*p* value	T value	R^2^ change
**Step 6**				**0.09**
Age	0.11 (− 0.17,0.38)	0.44	0.78	
Sex	− 0.42 (− 6.70,5.87)	0.90	− 0.13	
Verbal IQ	− 0.71 (− 1.30, − 0.12)	0.02	− 0.02	
Performance IQ	− 0.30 (− 1.00,0.40)	0.39	− 0.86	
Years with pain	0.03 (− 0.20,0.27)	0.77	0.29	
Medications Yes (ref)^a^	− 3.36 (− 9.91,3.17)	0.31	− 1.03	
Anticonvulsants yes (ref)	− 1.77 (− 8.29,4.75)	0.59	− 0.55	
Diagnosis FM (ref)	− 7.30 (− 13.25, − 1.35)	0.02	− 2.47	

^a^Anticonvulsants, antidepressants or opioids.

## Discussion

The main aim of this study was to compare patients with PNP to FM patients using four objective neuropsychological tests measuring executive functioning. In accordance with our first hypothesis, FM patients scored worse on measures of inhibitory control compared to PNP and healthy controls. However, this difference was not significant when controlled for age, sex and IQ.

In accordance with our second hypothesis, we found that patients with PNP had more errors than FM patients and healthy controls on the attention-demanding cued-recall task. When tested in a regression model, being in the PNP category remained significantly associated with worse cued recall (i.e. more mistakes) also when controlling for age, sex and IQ.

In the next step of analysis, the patients with PNP were compared head-to-head with FM patients, controlling for any other significant differences between the two pain categories. The association between the PNP group and cued recall remained significant even when controlling for years lived with pain, medication usage, as well as age, sex and IQ.

This indicates that the impairment was associated with the PNP category beyond any other significant differences between groups.

We see this result as complimentary to experimental data showing that pain only disrupts attentional and perceptual performance when interacting with a high load or demanding cognitive tasks^43,44^. The observed reduction in performance could therefore be tied to working memory function in PNP, as any diminishing of attention would disrupt the patients’ ability to maintain information in working memory^45^.

Our results could also stem from an attentional bias. A recent review showed that pain patients are better at recalling self-relevant material than neutral information, suggesting an attentional bias affecting recollection^2^. A popular theory is that this bias is due to a hypervigilance caused by living with pain. Hypervigilance referring to the idea that pain patients do not have a reduced attention, but rather a biased attention towards pain-related information driven by aversive conditioning and anxiety^12,23^. Differences in pain patients’ attentional bias and subsequent cued-recall, could be linked to differences in pain intensity^46^, or anxiety symptoms^47^, however, none of these proved to be significant in our analyses.

We would instead suggest a specific attentional deficiency or bias tied to persistent activation of peripheral nociceptors, as is the case in PNP. This is supported by pre-clinical data.

All though we are hesitant to the direct translation of results from animal models to humans, a recent rodent model of presumed PNP showed that repeated stimulation of peripheral nociceptive afferents greatly increased the disruptive effect from injury to the spinal nerve (L5) on visual attention^48^. The attentional disruption found in this study was tied to how demanding the task was for the subject studied.

In addition to this recent finding, impaired short-term plasticity or decreased neurogenesis in the hippocampus have previously been reported in animal models of presumed neuropathic pain^49^. This ties on to our results on the PAL test as a functional MRI study of participants performing the PAL test it was shown that the encoding phase of the task activated the hippocampus, and that extra-hippocampal areas show increased blood-oxygen-levels during PAL performance^50^. A specific impairment of attention in PNP patients affecting attention-demanding cued recall would then fit with the findings from pre-clinical data, suggesting hippocampus function as a potential mediator. As the PAL test has not been administered in previous studies of PNP, the current results could be the first indication of a specific disruption in PNP patients complaining about cognitive impairments.

Our ability to remember shapes and patterns is tied to the process of re-activating the same brain regions during two phases of memory, namely encoding and retrieval.

A neuroanatomical model has been proposed where the hippocampus binds neuronal changes in creating a bridge from encoding into memory^51^. The retrieval of this memory then relies on the hippocampus re-activating the neuronal network previously active in encoding. Speculating in a specific impairment of the hippocampus in PNP would fit with the current finding and the difference between groups may be tied to hippocampal function.

At the clinical level, this impairment corresponds to forgetfulness, the forgetfulness getting worse when the task is demanding and a delay separates the learning from the recall.

A practical consequence of our findings could be that PNP patients struggle with accurately remembering information given during complex presentations and meetings. The type of paired-associate learning tested in this study is required whenever you want to remember which objects you previously saw, along with the location where they were seen. It enables you to remember where you parked your car, but also impacts more complex pairing such as which password to use when you are entering a specific website or what budget post the speaker is referring to on his/her previous slide.

Newer studies have shown that if instructed, stroke patients with damage to a memory path can recruit alternative brain regions through top down learned strategies, improving memory performance^52^. The choice of using cued recall with patterns in this study was primarily because the test is not affected by previous learning. The presented stimuli are novel and changes for each administration, so the patient cannot learn from test to test. However, our results could have consequences for CBT and other psychological treatments of pain. Presuming that more data confirms our current finding, CBT could potentially be tailored to fit the specific impairment presented by PNP patients in this study. Should the PNP patient report cognitive impairments one could teach evidence-based strategies for improving encoding through attention training^53^.

Future investigations should include studying whether CBT or other psychological treatments are mediated by paired associative learning performance or whether specific executive impairments in different chronic pain conditions are improved by targeted cognitive training programs. It should also be an aim to investigate if a tailored attention training before receiving CBT could benefit patients with PNP, or even if attention training alone may be of benefit.

### Limitations

The participants in this study were selected from a large sample of patients attending a tertiary multi-disciplinary pain clinic, and represent a selection bias. Many patients will not experience cognitive impairments when living with pain, the current sample did, and the sample selected for this study should not be generalized to all patients with PNP or FM. However, the strict pain type identification procedure serving as the foundation of the study necessitated a large pool of participants to select from as both included pain conditions are not infrequently mislabeled by referring general practitioners.

## Conclusion

Patients with PNP appear to have a specific impairment when it comes to resource-demanding cued recall. This is the first study to show condition-specific impairment in this pain type using adequate and objective measures of executive function. The results demand further investigation and the rigorous methods applied should inform future research into specific impairments found in our sample.

## Supplementary Information

Supplementary Information.
