# Supplementary material for: Comparing objective cognitive impairments in patients with peripheral neuropathic pain or fibromyalgia
Source: Sci Rep. 2021 Jan 12;11:673. doi: 10.1038/s41598-020-80740-0 (PMC7803727; doi:10.1038/s41598-020-80740-0)
Supplement: Supplementary file 1 — Supplementary Information. [file 41598_2020_80740_MOESM1_ESM.docx]

# Appendix

**Description of tests and variables:**

***Executive component of inhibitory control:***

Stop Signal Task (SST)

The SST is a response inhibition test that measures a subject’s ability to inhibit a prepotent response. A white ring was presented in the middle of the screen in which an arrow appeared that pointed either to the left or to the right. There were two buttons on the screen and the subject was told to press the button on the side the arrow points to. Some trials (25%) required the subject to withhold the response and not to press the button, signaled by an auditory signal (a beep). The task used a tracking staircase function for the delay between the onset of the visual stimulus and the auditory stop signal (the stop signal delay, SSD): A failed stop trail reduced the subsequent delay by 50 milliseconds (ms) and a successful stop increased the delay by 50 ms. This method converged upon an SSD at which the subject successfully stopped approximately 50% of the time (SSD50, calculated using the second half of trials). The tests consisted of five blocks of 64 trials each. At the end of each block, a graph, representing the subject’s performance and a message to the subject (e.g., “Please try to go faster, but do keep stopping when you hear the beep”) was presented to the participants. The outcome variable used in the study was an estimate of the stop signal reaction time (SSRT) in milliseconds calculated by subtracting the SSD50 from the median reaction time on trials without stopping signal (lower results indicate higher performance). This outcome provides a measure of the speed of the inhibitory process (Logan & Cowan, 1984).

***Executive component of updating:***

Spatial Working Memory (SWM)

The SWM test assesses spatial working memory by measuring a subject’s ability to retain spatial information and to manipulate remembered items in working memory (Cambridge Cognition Limited, 2014). Three, four, six or eight colored boxes (depending on the stage) were displayed on the screen. Subjects had to use a process of elimination to find a blue token hidden inside the boxes. They had to touch each box in turn until one opened with a blue token inside. After finding a blue token in one of the boxes, this box would not contain a blue token again. This procedure continued until a blue token had been found in all boxes on the current screen. The test consisted of four practice trials and 12 trials that were recorded. Touching a box in which a blue token had already been found was an error. The pre-selected outcome was a component score reflecting the strategy participants´ used to avoid unnecessary errors.

***Executive component of flexibility:***

Intra-Extra Dimensional Shift (IED)

The IED is a computerized analog of the widely used Wisconsin Card Sorting Test and is a test of cognitive flexibility (Cambridge Cognition Limited, 2014). Four white-framed boxes were presented on the screen. In each trial, two stimuli (one correct and one incorrect) were presented in two of the boxes. These stimuli were based on two artificial dimensions, color-filled shapes and white lines, and were made up of either one. Feedback teaches the participant which stimulus is correct, and after six correct responses, the stimuli and/or rules are changed. Six consecutive correct responses within 50 trials are required to pass each stage; otherwise, the task ends. The rule for correct responding is modified at each stage to dissociate different aspects of cognitive flexibility. The shifts in correct stimuli are initially intra-dimensional (e.g., within the shape dimension) and then later extra-dimensional, requiring a category shift (e.g., from the shape dimension to the line dimension). The pre-selected outcome was IED total errors, a composite of the number of completed stages and the number of errors made.

***Attention-demanding cued recall***

Paired Associates Learning (PAL)

The PAL test is a cued recall test that assesses visual memory and new learning (Cambridge Cognition Limited, 2014). Six or eight white boxes (depending on the stage) were displayed on the screen and opened in a randomized order. Depending on the stage, the test presents boxes containing one, two, three, six or eight patterns. After all boxes had been opened the patterns shown in the boxes were displayed in the middle of the screen, one at a time. The subject had to touch the box in which the pattern was originally located. At each stage the subjects were given up to ten attempts to get all the locations correct. If the subject could not complete a stage correctly, the test terminated. The pre-selected outcome was performance on the hardest stage, total errors 8 shapes adjusted. Total errors 8 shapes adjusted report the numbers of errors made on the last, most difficult stage of the PAL test.

**Medication:** Participants reported daily medication usage upon inclusion and this was controlled with the list of medications provided by the general practitioner and any information given during the interview by the pain specialist. Pharmaceuticals were classified by a consensus given by expert physicians as: ”Opioids” ”Anticonvulsants” or ”Antidepressants” based on their active pharmacological ingredients. Morphine equivalents were calculated using a standard calculator provided by the Centers for Disease Control ([www.cdc.gov/drugoverdose/prescribing/guideline.html](http://www.cdc.gov/drugoverdose/prescribing/guideline.html)).

**Patient reported variables:**

*Usual pain intensity* was measured with one item asking the participant to rate how intense their pain usually was felt the last week. The item is scored from 0 to 10, with 0 representing **“**no pain**”** and 10 the **“**worst pain imaginable**”**.

*Pain bothersomeness* was measured with one item asking the participant to rate how bothersome their pain was in the previous week. The item is scored from 0 to 10, with 0 representing **“**not at all**”** and 10 the **“**worst imaginable**”**. These measures have been validated in a large Norwegian population study (Jacobsen, Eggen, Mathiesen, Wilsgaard, & Njølstad, 2012).

*Fatigue* was measured through the Chalder Fatigue Scale (Chalder et al., 1993), which is a self-rating scale developed to measure the severity of fatigue. It is an 11-item scale and is found to be both reliable and valid (Chalder et al., 1993), also in Norwegian (Loge, Ekeberg, & Kaasa, 1998).

*Work status* was defined as participants working or not working using two items; “Do you currently work?” and “Do you receive any of the following benefits?” (“Disability pension” “Sick leave benefits” “Workers compensation”) as well as any other benefits pertaining to being out of work. Work status 0 (employed) was then calculated as answering “yes” to item 1 and “no” to item 2. Participants received a score of 1 (not working) if reporting “no” on item 1 and/or “yes” on item 2 (Granan et al., 2019).

A modified *Oswestry Disability Index* (ODI) was used to assess function. The original ODI has 10 items concerning back pain and different activities of daily life (personal care, lifting, walking, sitting, standing, sleeping, sexual life, social life and travelling). Each item is scored from 0 to 5, with higher values representing more disability. ODI has high reliability and validity [14], and is validated in Norwegian [15]. The modified ODI used in the OPR is identical to the original ODI with one exception; the word “back” is deleted, and this deletion only occurs once, in the introduction of the form (Granan et al., 2019).

The *Insomnia Severity Index* (ISI) was used to assess the levels of insomnia symptoms (29). The ISI is a seven-item self-report questionnaire measuring the nature, severity and impact of insomnia symptoms the past two weeks. The items are: 1) difficulty falling asleep, 2) difficulty maintaining sleep, 3) early morning awakenings, 4) satisfaction/dissatisfaction with sleep pattern, 5) interference of sleep problems with daily functioning, 6) sleep problems being noticeable by others and 7) levels of distress/worry caused by the sleep problems. Each item is rated by using a 5-point Likert scale (e.g., 0 = no problem; 4 = very severe problem) giving a total score ranging from 0 to 28. The ISI has a very good reliability and validity [29].

**Tables:**

Table A1. The executive functioning outcome PAL analyzed using a multivariate linear regression model. The category and its association with this outcome were controlled for age, sex and all other variables showing significant differences between categories. These variables were then added in steps.

| **Steps** |  | **B** | **Std. Error** | **T-value** | **P-value** | **95 % CI**  **Lower Bound** | **95 % CI**  **Upper Bound** |
| --- | --- | --- | --- | --- | --- | --- | --- |
| 1 | (Constant) | 9.82 | 7.67 | 1.28 | 0.21 | -5.53 | 25.18 |
|  | Age | 0.07 | 0.13 | 0.52 | 0.61 | -0.19 | 0.33 |
|  | Sex | 0.52 | 3.28 | 0.16 | 0.88 | -6.05 | 7.08 |
| 2 | (Constant) | 31.83 | 10.67 | 2.98 | 0.00 | 10.45 | 53.22 |
|  | Age | 0.06 | 0.14 | 0.47 | 0.64 | -0.21 | 0.34 |
|  | Sex | 0.62 | 3.10 | 0.20 | 0.84 | -5.60 | 6.83 |
|  | Verbal IQ | -0.73 | 0.30 | -2.43 | 0.02 | -1.32 | -0.13 |
|  | Performance IQ | -0.30 | 0.34 | -0.86 | 0.39 | -0.98 | 0.39 |
| 3 | (Constant) | 31.81 | 10.75 | 2.96 | 0.01 | 10.26 | 53.35 |
|  | Age | 0.08 | 0.14 | 0.54 | 0.59 | -0.21 | 0.36 |
|  | Sex | 0.41 | 3.15 | 0.13 | 0.90 | -5.91 | 6.73 |
|  | Verbal IQ | -0.75 | 0.30 | -2.46 | 0.02 | -1.35 | -0.14 |
|  | Performance IQ | -0.25 | 0.36 | -0.70 | 0.49 | -0.97 | 0.47 |
|  | Years with pain | -0.05 | 0.11 | -0.48 | 0.63 | -0.28 | 0.17 |
| 4 | (Constant) | 31.57 | 10.77 | 2.93 | 0.01 | 9.96 | 53.18 |
|  | Age | 0.09 | 0.14 | 0.66 | 0.51 | -0.19 | 0.38 |
|  | Sex | 0.79 | 3.19 | 0.25 | 0.81 | -5.60 | 7.19 |
|  | Verbal IQ | -0.74 | 0.30 | -2.44 | 0.02 | -1.35 | -0.13 |
|  | Performance IQ | -0.23 | 0.36 | -0.63 | 0.53 | -0.95 | 0.49 |
|  | Years with pain | -0.06 | 0.11 | -0.56 | 0.58 | -0.29 | 0.16 |
|  | Medications Yes (ref) + | -2.37 | 2.72 | -0.87 | 0.39 | -7.81 | 3.08 |
| 5 | (Constant) | 31.94 | 10.96 | 2.92 | 0.01 | 9.96 | 53.92 |
|  | Age | 0.09 | 0.14 | 0.63 | 0.53 | -0.20 | 0.38 |
|  | Sex | 0.89 | 3.24 | 0.27 | 0.79 | -5.60 | 7.38 |
|  | Verbal IQ | -0.75 | 0.31 | -2.44 | 0.02 | -1.37 | -0.13 |
|  | Performance IQ | -0.23 | 0.36 | -0.64 | 0.53 | -0.96 | 0.50 |
|  | Years with pain | -0.07 | 0.12 | -0.58 | 0.56 | -0.30 | 0.16 |
|  | Medications Yes (ref) + | -1.84 | 3.36 | -0.55 | 0.59 | -8.58 | 4.90 |
|  | Anticonvulsants yes (ref) | -0.92 | 3.38 | -0.27 | 0.79 | -7.71 | 5.87 |
| 6 | (Constant) | 45.10 | 11.74 | 3.84 | 0.00 | 21.53 | 68.67 |
|  | Age | 0.11 | 0.14 | 0.78 | 0.44 | -0.17 | 0.38 |
|  | Sex | -0.42 | 3.13 | -0.13 | 0.90 | -6.71 | 5.87 |
|  | Verbal IQ | -0.71 | 0.29 | -0.02 | 0.02 | -1.30 | -0.12 |
|  | Performance IQ | -0.30 | 0.35 | -0.86 | 0.39 | -1.00 | 0.40 |
|  | Years with pain | 0.03 | 0.12 | 0.29 | 0.77 | -0.20 | 0.27 |
|  | Medications Yes (ref) + | -3.36 | 3.26 | -1.03 | 0.31 | -9.91 | 3.19 |
|  | Anticonvulsants yes (ref) | -1.77 | 3.25 | -0.55 | 0.59 | -8.29 | 4.75 |
|  | Diagnosis (Fibromyalgia - ref) | -7.30 | 2.96 | -2.47 | 0.02 | -13.25 | -1.35 |
| + Anticonvulsants, antidepressants or opioids | | | | | | | |

Table A2. The executive functioning outcome SST analyzed using a multivariate linear regression model. The category and its association with this outcome were controlled for age, sex IQ and group categories (FM, PNP and HV). These variables were then added in steps.

| **Steps** |  | **B** | **Std. Error** | **P-value** | **T-value** | **95 % CI**  **Lower Bound** | **95 % CI**  **Upper Bound** |
| --- | --- | --- | --- | --- | --- | --- | --- |
| 1 | Age | 1.67 | 0.62 | 0.01 | 2.68 | 0.42 | 2.91 |
|  | Sex | -0.79 | 16.12 | 0.96 | -0.05 | -33.05 | 31.48 |
| 2 | Age | 1.61 | 0.64 | 0.02 | 2.51 | 0.33 | 2.90 |
|  | Sex | 0.03 | 15.99 | 1.00 | 0.00 | -31.98 | 32.04 |
|  | Verbal IQ | -1.96 | 1.39 | 0.17 | -1.41 | -4.75 | 0.83 |
|  | Performance IQ | -0.93 | 1.50 | 0.54 | -0.62 | -3.93 | 2.06 |
| 3 | Age | 1.53 | 0.64 | 0.02 | 2.39 | 0.25 | 2.81 |
|  | Sex | 5.63 | 16.30 | 0.73 | 0.35 | -27.03 | 38.28 |
|  | Verbal IQ | -1.87 | 1.38 | 0.18 | -1.35 | -4.64 | 0.90 |
|  | Performance IQ | -1.04 | 1.48 | 0.49 | -0.70 | -4.01 | 1.93 |
|  | Group (HV is ref; FM:2 PNP:3) | -19.47 | 13.46 | 0.15 | -1.45 | -46.43 | 7.48 |

Table A3. The executive functioning outcome SWM analyzed using a multivariate linear regression model. The category and its association with this outcome were controlled for age, sex IQ and group categories (FM, PNP and HV). These variables were then added in steps.

| **Steps** |  | **B** | **Std. Error** | **P-value** | **T-value** | **95 % CI**  **Lower Bound** | **95 % CI**  **Upper Bound** |
| --- | --- | --- | --- | --- | --- | --- | --- |
| 1 | Age | 0.18 | 0.08 | 0.02 | 2.35 | 0.03 | 0.33 |
|  | Sex | -3.64 | 1.96 | 0.07 | -1.86 | -7.56 | 0.27 |
| 2 | Age | 0.16 | 0.08 | 0.04 | 2.13 | 0.01 | 0.31 |
|  | Sex | -3.42 | 1.86 | 0.07 | -1.84 | -7.15 | 0.30 |
|  | Verbal IQ | -0.34 | 0.17 | 0.05 | -2.05 | -0.68 | -0.01 |
|  | Performance IQ | -0.23 | 0.18 | 0.21 | -1.26 | -0.58 | 0.13 |
| 3 | Age | 0.15 | 0.08 | 0.05 | 1.97 | 0.00 | 0.30 |
|  | Sex | -2.78 | 1.92 | 0.15 | -1.45 | -6.63 | 1.06 |
|  | Verbal IQ | -0.33 | 0.17 | 0.05 | -2.00 | -0.67 | 0.00 |
|  | Performance IQ | -0.24 | 0.18 | 0.19 | -1.33 | -0.59 | 0.12 |
|  | Group (HV is ref; FM:2 PNP:3) | -2.02 | 1.61 | 0.22 | -1.26 | -5.25 | 1.21 |

Table A4. The executive functioning outcome IED analyzed using a multivariate linear regression model. The category and its association with this outcome were controlled for age, sex IQ and group categories (FM, PNP and HV). These variables were then added in steps.

| **Steps** |  | **B** | **Std. Error** | **P-value** | **T-value** | **95 % CI**  **Lower Bound** | **95 % CI**  **Upper Bound** |
| --- | --- | --- | --- | --- | --- | --- | --- |
| 1 | Age | 0.18 | 0.08 | 0.02 | 2.35 | 0.03 | 0.33 |
|  | Sex | -3.64 | 1.96 | 0.07 | -1.86 | -7.56 | 0.27 |
| 2 | Age | 0.16 | 0.08 | 0.04 | 2.13 | 0.01 | 0.31 |
|  | Sex | -3.42 | 1.86 | 0.07 | -1.84 | -7.15 | 0.30 |
|  | Verbal IQ | -0.34 | 0.17 | 0.05 | -2.05 | -0.68 | -0.01 |
|  | Performance IQ | -0.23 | 0.18 | 0.21 | -1.26 | -0.58 | 0.13 |
| 3 | Age | 0.15 | 0.08 | 0.05 | 1.97 | 0.00 | 0.30 |
|  | Sex | -2.78 | 1.92 | 0.15 | -1.45 | -6.63 | 1.06 |
|  | Verbal IQ | -0.33 | 0.17 | 0.05 | -2.00 | -0.67 | 0.00 |
|  | Performance IQ | -0.24 | 0.18 | 0.19 | -1.33 | -0.59 | 0.12 |
|  | Group (HV is ref; FM:2 PNP:3) | -2.02 | 1.61 | 0.22 | -1.26 | -5.25 | 1.21 |
